# Integrated molecular characterization of adult soft tissue sarcoma for therapeutic targets

**DOI:** 10.1186/s12881-018-0722-6

**Published:** 2018-12-31

**Authors:** Jihyun Kim, June Hyuk Kim, Hyun Guy Kang, Seog Yun Park, Jung Yeon Yu, Eun Young Lee, Sung Eun Oh, Young Ho Kim, Tak Yun, Charny Park, Soo Young Cho, Hye Jin You

**Affiliations:** 1 Clinical Genomic Analysis Branch, Research Institute, National Cancer Center, 323 Ilsan-ro, Ilsandong-gu, Goyang, Gyeonggi 10408 South Korea; 2 Orthopaedic Oncology Clinic, Hospital, National Cancer Center, Goyang, Gyeonggi 10408 South Korea; 3 Department of Cancer Biomedical Science, NCC-GCSP, National Cancer Center, Goyang, Gyeonggi 10408 South Korea; 4 Division of Pathology, Hospital, National Cancer Center, Goyang, Gyeonggi 10408 South Korea; 5 Translational Research Branch, Research Institute, National Cancer Center, Goyang, Gyeonggi, 10408 South Korea; 6 Rare Cancer Branch, Research Institute, National Cancer Center, Goyang, Gyeonggi 10408 South Korea

**Keywords:** Complex karyotype sarcoma, Molecular characterization, PDGFRA, CDK4 and RB1

## Abstract

**Background:**

Several studies have investigated the molecular drivers and therapeutic targets in adult soft tissue sarcomas. However, such studies are limited by the genomic heterogeneity and rarity of sarcomas, particularly in those with complex and unbalanced karyotypes. Additional biomarkers are needed across sarcoma types to improve therapeutic strategies. To investigate the molecular characteristics of complex karyotype sarcomas (CKSs) for therapeutic targets, we performed genomic profiling.

**Results:**

The mutational landscape showed that TP53, ATRX, and PTEN genes were highly mutated. CKS samples were categorized into three groups based on copy number variations that were associated with CDK4 and RB1 signatures. Integrated analysis of genomic and transcriptomic data revealed several pathways related to PDGFR, which could be a strategic target for anti-sarcoma therapy.

**Conclusions:**

This study provides a detailed molecular classification of CKSs and proposes several therapeutic targets. Targeted or combinational therapies for treating CKS should be considered before chemotherapy.

**Electronic supplementary material:**

The online version of this article (10.1186/s12881-018-0722-6) contains supplementary material, which is available to authorized users.

## Background

Soft tissue sarcomas (STSs) are rare cancers, comprising less than ~ 1% of all cancers, and can arise anywhere in the body. STSs are classified according to the morphology of the tissue they resemble. According to the World Health Organization classification reported in 2013, STSs include more than 50 histologic subtypes with diverse clinical behaviors, responses to chemotherapy, and overall outcomes. Among them, liposarcoma (LPS), undifferentiated sarcoma (US), leiomyosarcoma (LMS), myxofibrosarcoma (MFS), and synovial sarcoma (SS) are the most common types in adults. The outcomes vary considerably by sarcoma subtype, distinct molecular characteristics, and behavioral features [1]. STSs, grouped broadly according to molecular complexity, are classified into two groups: simple karyotype sarcoma with balanced translocation and complex karyotype sarcoma (CKS) without aberrant dislocation [2]. Translocation-associated sarcomas, most represented by LPS and SS, account for 20–30% of all sarcomas [3]. Although targeted therapies against fusion genes have not been successful, it has been postulated that translocation variants in sarcoma are predictive of patient outcome. CKSs comprise the largest category of STSs, which includes undifferentiated pleomorphic sarcoma (UPS; previously known as malignant fibrous histiocytoma), LMS, and MFS, in order of frequency [4]. CKS subtypes are heterogeneous, unstable, and profoundly altered in their genomic copy number [2].

Several additional genes have been identified and characterized since Barretina et al. first defined genes involved in STSs based on subtype-specific genomic alterations [5]. For example, *NF1* tumor suppressor is mutated or deleted in several sarcoma subtypes. *CDK4* and *YEATS4* are related to cell proliferation. *PI3KCA* mutations are associated with poor clinical outcome. Recently, our understanding of STS has been strengthened by TCGA reports [6], and copy-number alterations in adult STSs show clear clustering. However, previous studies discussed tumor type specific molecular features and did not provide molecular reclassification for therapeutic target. The rarity of these cancers hinders progress in developing potential drug therapies. To develop anticancer strategies against STSs, further information is required, particularly in those with complex karyotypes and pathways for tumor progression.

Here we provide molecular reclassification by characterizing the alterations of copy number and mRNA expression, and present potential therapeutic target for clinical application irrespective of histological types. We used 14 fresh frozen samples for whole exome sequencing (WXS) and whole transcriptome sequencing. Comparing the copy number alterations and mRNA profiles, we found that three molecular types were easily distinguishable from CKS patients. CDK4 amplification and RB1 deletion were seen in patterns of genomic damage and PDGFRA profiles could be used to clearly separate samples. We identified PDGFRA target drug disruption in CDK4 amplification group. In conclusion, clusters were closely related to potential therapeutic targets as for anti-cancer drug strategy in STS.

## Methods

### Patient samples

This study was approved by the Ethics Review Board of the National Cancer Center, Korea (IRB No.: NCC2017–0062). Written informed consent was obtained from all patients before tissue acquisition, and all samples were stored according to the principles of the Declaration of Helsinki. Tumor materials were obtained from the National Cancer Center Biobank. Frozen specimens from 14 adult patients with STS were obtained from the Biobank; 13 patients were Korean, and one patient was Russian. Table 1 lists the clinicopathologic features of the patients and tumors. Frozen tissues were sliced and stained with hematoxylin and eosin (H&E). To ensure adequate tumor cell density, a pathologist specializing in sarcomas reviewed the stained slides. Microdissection was performed to obtain the tumor and adjacent non-tumor cells from H&E-stained frozen sections. Genomic DNA and RNA were extracted using DNeasy and RNeasy Blood and Tissue Kits (Qiagen, Hilden, Germany), respectively.

**Table 1:** Summary of clinical and pathologic information in soft tissue sarcoma.

Total No. affected individual	14
Age (mean ± S.D. (range))	53 ± 30 (23–75)
Gender	
Female	5 (35.7%)
Male	9 (64.3%)
Tumor size	
0-5 cm	5 (35.7%)
5-10 cm	6 (42.9%)
10-15 cm	3 (21.4%)
Grade (FNCLCC)	
I	1 (7.1%)
II	4 (28.6%)
III	9 (64.3%)
Primary site	
Upper extremity	2 (14.3%)
Lower leg	2 (14.3%)
Thigh	9 (64.3%)
Pelvis	1 (7.1%)
Stage at time of sample procurement	
Primary	9 (64.3%)
Local recurrence	3 (21.4%)
Distant recurrence	2 (14.3%)
Histology	
Myxofibrosarcoma	3 (21.4%)
Leiomyosarcoma	5 (35.7%)
Undifferentiated sarcoma	6 (42.9%)
Undifferentiated pleomorphic sarcoma	4 (28.6%)
Undifferentiated round cell sarcoma	1 (7.1%)
Undifferentiated spindle cell sarcoma	1 (7.1%)
Pre-operative Therapy	
None	11 (78.6%)
Pre-operative Chemotherapy	2 (14.3%)
Pre-operative Radiation-Therapy	0 (0.0%)
Both	1 (7.1%)
Median follow-up	42.29 months
Time to relapse	
Local recurrence	2.0 months
Distant recurrence	6.1 months

### Exome sequencing and mutation calling

WXS was captured using the SureSelectXT library kit and performed on Illumina’s Hiseq2500 platform for paired-ends. Before alignment, poor quality read bases were cut off using Trimmomatic v0.36 [7]. Exome sequencing data from paired tumor and normal samples were aligned independently with the hg19 version of the human reference using BWA v0.7.13, and duplicated reads were removed using Picard v2.1.1. Mapped BAM files were processed according to GATK [8] best practices, including insertion/deletion (indel) realigning, mate fixing, and recalibration. Somatic mutations were called using MuTect [9]. To improve accuracy, data from dbSNP Build 138 and COSMIC v76 were supplied as parameters to MuTect. Somatic indels were identified using Strelka v1.0.14 [10]. We applied Oncotator [11] to annotate the mutation impact of somatic variants that called the above processes. Raw DNA sequencing data have been deposited in the European Nucleotide Archive (ENA) under primary accession number PRJEB23898.

### Mutation signature analysis and microsatellite instability (MSI) clustering

To investigate the mutational process, we selected single-nucleotide alterations and converted them into the six classes of base substitution (C > A, C > G, C > T, T > A, T > C, and T > G) [12]. Non-negative matrix factorization (NMF) clustering was applied to detect mutation signatures from called somatic mutation using R package SomaticSignatures.

To identify the MSI status, we collected 42 MSI-high signatures across 18 cancer types from a previous report [13]. Next, we performed unsupervised consensus clustering from 42 gene expression profiles using R package NMF.

### Copy number analysis

Copy number variants from WXS were estimated using EXCAVATOR2 [14]. We ran each sample of BAM files through the EXCAVATOR2 pipeline and identified the aberrant regions that were highly enriched with copy number gains/losses in all 14 samples using GISTIC2.0 [15]. The X and Y chromosomes were excluded from analysis. The copy number data from 206 patients were downloaded from Level 4 available data archives on the TCGA Data Portal website.

### RNA sequencing alignment and quantification

RNA sequencing was performed on Illumina’s Hiseq2500 sequencing platform using TruSeq libraries for paired-end sequencing. All data were processed using the International Cancer Genome Consortium pipelines. After cutting bases from poor quality reads using Trimmomatic v0.36 [7], a two-pass method was employed, with STAR [16] used for read alignment and RSEM v1.3.0 [17] used for gene expression quantification. RSEM was used to estimate the gene expression values as transcripts per million. Raw RNA sequencing data have been deposited in the ENA (primary accession number PRJEB24352).

### Network and gene set enrichment analysis (GSEA)

For human PPI network analyses, we integrated information from available public databases, including HPRD, BioGRID, IntAct, MINT, and Reactome. A total of 136,489 interactions among 14,215 human proteins were prepared. All gene networks were visualized using Cytoscape [18]. Overrepresented pathways and module analysis of custom networks were identified using Cytoscape ReactomeFI and MCODE plug-in software, respectively [18]. GSEA was performed in R package GSVA, with the KEGG pathway used as the background pathway database [19]. Thresholds for significant pathways were determined as: FDR < 0.05; GSEA *P* < 0.05.

### Drug response analysis

Using the Cancer Therapeutics Response Portal v2, we found that the *PDGFRA* gene was targeted by six drugs, including axitinib, Ki8751, lenvatinib, masitinib, nintedanib, and sunitinib. To investigate the drug response in sarcoma cell lines, drug response data were downloaded from a study by Iorio et al. [20], and genomic alteration data were downloaded from the Cancer Cell Line Encyclopedia (CCLE) [21]. We investigated drug concentrations that reduced viability by 50% (IC_50_) and the area under the dose-response curve values to determine drug target sensitivity. We detected four cell lines (G402, A204, TE441T, and G401) that had no amplification of *CDK4* (copy number score < 0.1) and displayed positive gene expression of *PDGFRA*, and seven cell lines (SKUT1, MESSA, GCT, SKLMS1, RH41, RH18, and RH30) that had *CDK4* amplification (amp) (> 0.1) and positive expression of *PDGFRA*. To determine the applicability of cancer therapies, we analyzed the following subgroups: 1) cell lines of the RB1 del type (SKUT1, MESSA, TE617T, S117, and RKN), which showed low gene expression of *CDK4*, *PDGFRA*, and *RB1*; and 2) cell lines of the *CDK4* amp type (RH41 and G401), which showed high expression of *CDK4*, *PDGFRA*, and *RB* genes*.*

## Results

### Patient characteristics

Tumor and germline DNA/RNA samples were obtained from 14 patients with a median age of 53 years (range 23–75 years). There were 9 male and 5 female patients. Thirteen patients were Korean and one patient was Russian. Tumor samples consisted of six Undifferentiated sarcomas (US; 4 UPS, 1 undifferentiated round cell sarcoma, 1 undifferentiated spindle sarcoma), five LMS, and three MFS. Tumors were mostly high-grade (grade II and III, 93%), and one patient had low-grade (grade I, according to the FNCLCC grading system) disease. Three samples were obtained from recurred tumor and two were from metastatic tumor. All metastatic samples were obtained from the soft tissue extremities. 3 of the 14 patients had chemotherapy or radiotherapy before tissue acquisition. One patient with recurred leiomyosarcoma had chemotherapy and the patient with recurred undifferentiated spindle cell sarcoma had chemotherapy and radiation therapy, which performed 6 years before surgery for recurrence in both patients. The other patient with recurred leiomyosarcoma had chemotherapy for double malignancy (acute myeloblastic leukemia), which performed 3 years before surgery. Table 1 lists all clinical characteristics.

### Molecular landscape of three CKSs

**Fig. 1:**
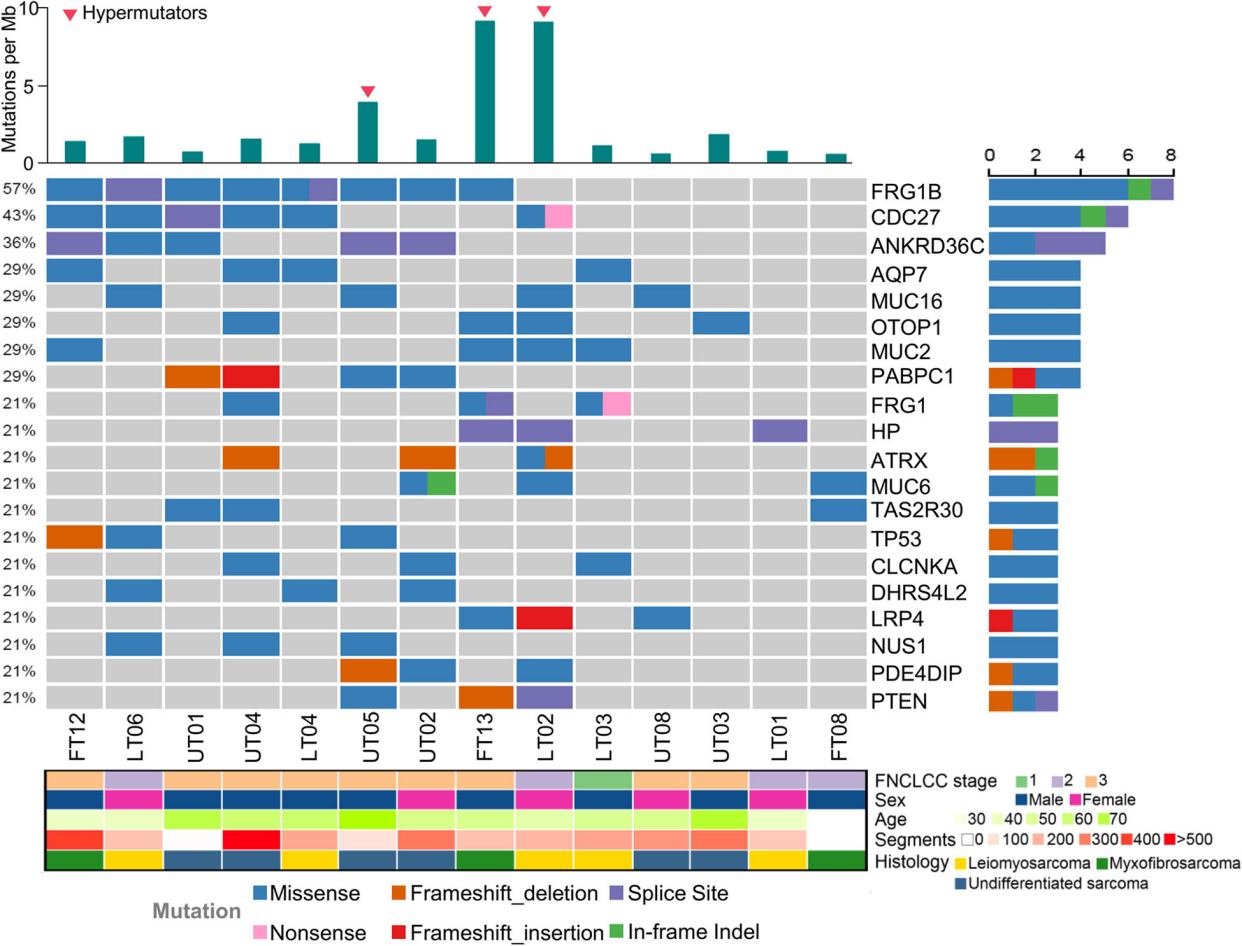
Molecular landscape of complex karyotype sarcomas (CKSs). Data plots of the clinical and molecular features in CKS samples from 14 patients. Top panel: frequency of mutations per Mb; middle panel: mutation plot of 20 genes ordered by mutation frequency; right panel: mutation frequency of selected 20 genes; lower panel: sarcoma grade, patient sex, patient age, number of genomic segments, and sarcoma type. Color keys at right and bottom. FT, Myxofibrosarcoma; LT, Leiomyosarcoma; UT, Undifferentiated sarcoma

We investigated the somatic molecular landscape across three CKS types. In total, we identified 1819 somatic truncation variants (1394 missenses, 220 frameshift indels, 33 in-frame indels, 62 nonsenses, and 110 splice sites). Median coverage was 62.11× in normal and 116.03× in tumor samples. We found that *FRG1B, CDC27, TP53, ATRX,* and *PTEN* were recurrent across three CKS types. Fig. 1 lists the most frequently mutated (top 20) genes. *FRG1B* and *CDC27* were the most highly mutated genes in CKSs (57 and 42%, respectively). *TP53*, *ATRX,* and *PTEN* were recurrently mutated (21.4%), consistent with a previous report on STSs [6]. The median mutation frequency was 2.38 per Mb (range 0.51–8.76 per Mb). The hypermutation samples were classified using mutation rates; three samples (UT05, FT13, and LT02; Fig. 1) had mutation rates exceeding 1.5 times the length of the interquartile range from the 75th percentile [22]. *PTEN* mutations were enriched in hypermutators (100%; *P* = 0.003, Fisher’s exact test). There was no co-occurrence or mutual exclusiveness when comparing the clinical characteristics (age, disease subtype, tumor degree) in those with or without identified genetic aberrations.

### MSI signatures in CKS

**Fig. 2:**
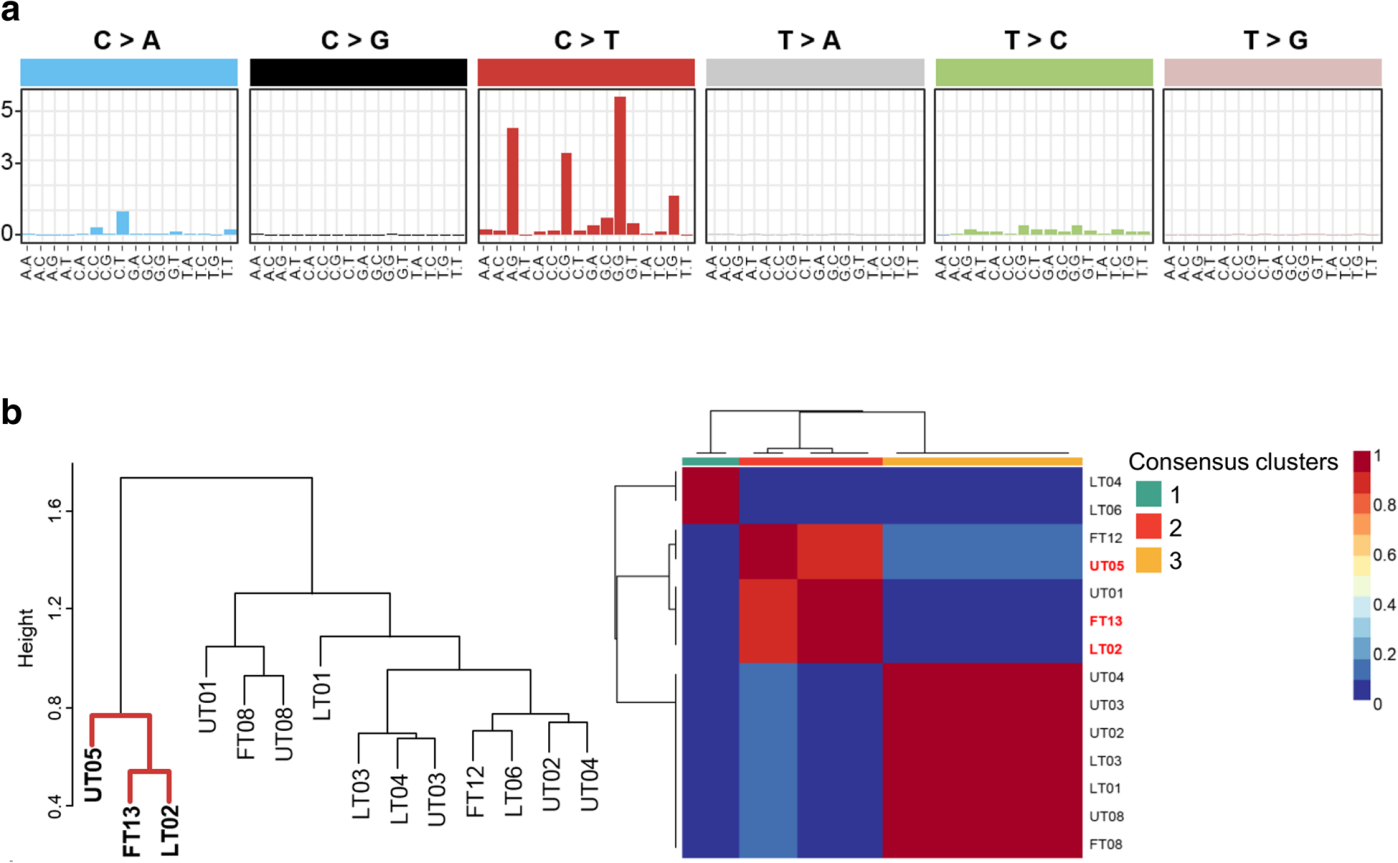
Mutation signature and microsatellite instability (MSI) clustering. **a** Contribution of the different types of substitutions in all patients. **b** The dendrogram was determined using the frequency of substitutions motif by hierarchical clustering. Red lines indicate clusters of hypermutators**.** Consensus plot for three non-negative matrix factorization (NMF) clusters (10 runs) by mRNA expression across the 42 MSI signature genes. Red color indicates hypermutators

**Fig. 3:**
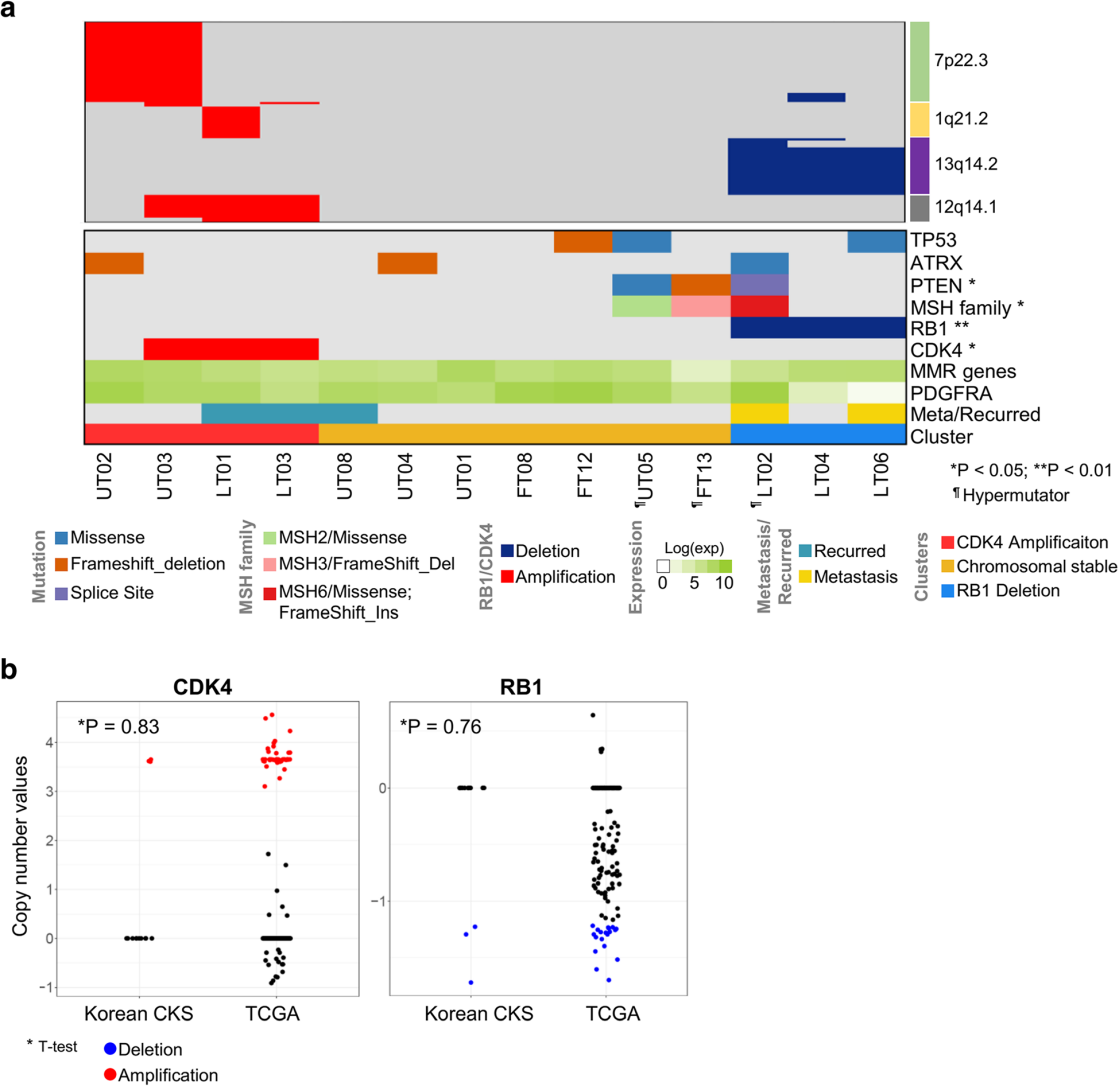
Somatic copy number alteration (SCNA) clustering and molecular subtypes. **a** Hierarchical clustering using the copy number profiles of focal regions from GISTIC2. Right bars indicate significant peak regions (7p22.3, 1q21.2, 13q14.2, and 12q14.1) from GISTIC2. Bottom panel: significantly mutated genes mentioned previously (*TP53*, *ATRX*, *PTEN*, and *MSH* family); copy number alterations in *CDK4* (red) or *RB1* (blue); gene expression profiles with DNA mismatch repair genes (*MSH2*, *MSH3*, *MSH6*, and *PMS2*) and *PDGFRA*; patient tumor status; cluster with SCNA status. *P*-values were calculated by Fisher’s exact test. **b** Comparison with copy number values of *CDK4*/*RB1* in focal regions from CKSs and TCGA data. Red dots indicate patients with *CDK4* amplification (copy number values > 2) and blue dots indicate patients with *RB1* deletion (copy number values < − 1.2). P-values were calculated by *t*-test

Mutation signatures are a catalogue of somatic mutations that can be used to find hidden patterns for somatic mutations in human cancer. We observed a high mutational burden of increased C > T and/or C > A mutations at NpCpG (Fig. 2a). Alexandrov et al. designated this substitution pattern as mutational signature 6 [12]. Mutation signature 6, attributed to DNA mismatch repair, is found in microsatellite unstable tumors. Two distinct clusters with mutational frequency were revealed using hierarchical clustering (Fig. 2b). One cluster was dominated by three samples, annotated as hypermutators (UT05, FT13, and LT02). We examined the mRNA expression of 42 high-level MSI (MSI-h) signature genes to predict the MSI status using mRNA expression; three sub-clusters were identified by non-negative matrix factorization (NMF) algorithm. Of these sub-clusters, MSI-h signature genes were highly expressed in cluster 2, comprising hypermutators (*P* = 0.19, F-test; Additional file 1: Figure S1). Additionally, the hypermutators contained somatic mutations in DNA mismatch repair genes such as *MSH2*, *MSH3*, and *MSH6*, and showed a slight association with the downregulation of these genes (Fig. 3a).

### CKS characterization by somatic copy number alteration

To identify the molecular characteristics of CKSs, WXS was used to predict the somatic copy number alteration (SCNA). DNA copy number alteration is the primary mechanism in sarcomagenesis [2]. Significantly enriched alterations in CKSs were identified using GISTIC [15]. In this study, we found amplifications of 1q21.2, 7p22.3, and 12q14.1, which occur frequently in sarcomas [23–25], and represent a prognostic factor for adverse outcomes in a variety of cancer types [26]. Also, deletions were found in 11q24.2, 12p13.31, and 13q14.2, reported previously in sarcoma [27] (Additional file 1: Figure S2). We classified focal SCNAs: 63 regions were amplified (copy number value > 2.0) and 25 regions were deleted (copy number value < − 1.2). Unsupervised hierarchical clustering was performed according to 88 focal SCNA regions; three clusters were identified (Fig. 3a). All sarcoma types were scattered among the clusters. The first cluster contained four patients with LMS and US. The second cluster contained seven patients with MFS and US. The third cluster had three patients with LMS. The first cluster was enriched with *CDK4* amplification (75%; *P* = 0.032, Fisher’s exact test), which has an oncogenic role in sarcomagenesis [5]. Additionally, patients who were diagnosed with a recurrence had tumors that were enriched with *CDK4* amplification (66.7%; Fig. 3a). According to the TCGA sarcoma data, *CDK4* amplification and recurrence showed co-occurrence (*P* = 1.60e-05, Chi-square test). The second cluster was enriched with the *RB1* deletion (100%; *P* = 0.002, Fisher’s exact test), which accelerates additional cancer gene mutations in sarcomagenesis [28]. Patients with metastasis had tumors that were enriched in the *RB1* deletion (66%; Fig. 3a). Although *RB1* loss and metastasis showed no correlation in the TCGA sarcoma data, a strong association of *RB1* deletion with metastasis was reported previously in human and mice [29, 30]. The third cluster showed chromosomal stability. UT05 and FT13 in the third cluster were classified as MSH-h samples by mutation signature and MSI gene expression patterns. We found three molecular groups in the CKSs based on SCNA, including the *CDK4* amplification enriched group (*CDK4* amp group), *RB1* deletion enriched group (*RB1* del group), and stable chromosome group (CS group) except for patients who observed MSI-h and high mutation rates. As expected, these clusters based on SCNA similarly identified in 206 TCGA sarcoma data (Additional file 1: Figure S3).

### PDGFRA pathway disarrangement in CKS

Correlation analysis of the three groups revealed a relationship between gene copy number and expression profiles. Analysis of genes in six significant peaks from GISTIC revealed that 26 genes, including *CDK4* and *RB1*, had a positive correlation with copy number values and gene expression. Using the human protein-protein interaction (PPI) network, we constructed sub-networks with 558 genes and 11,643 interactions that included the 26 genes and their first neighbors (Additional file 1: Figure S4 a,b). Then, the biological pathways were examined by using Cytoscape ReactomeFI plug-in. Cell cycle:G1/S check point (FDR = 7.19e-03), PDGF receptor signaling network (FDR = 9.93e-03), RB tumor suppressor/checkpoint signaling in response to DNA damage (FDR = 0.02), and signaling by the TGF-beta receptor complex (FDR = 0.03) were significantly enriched in these genes but no amplification of *CDK4*.

**Fig. 4:**
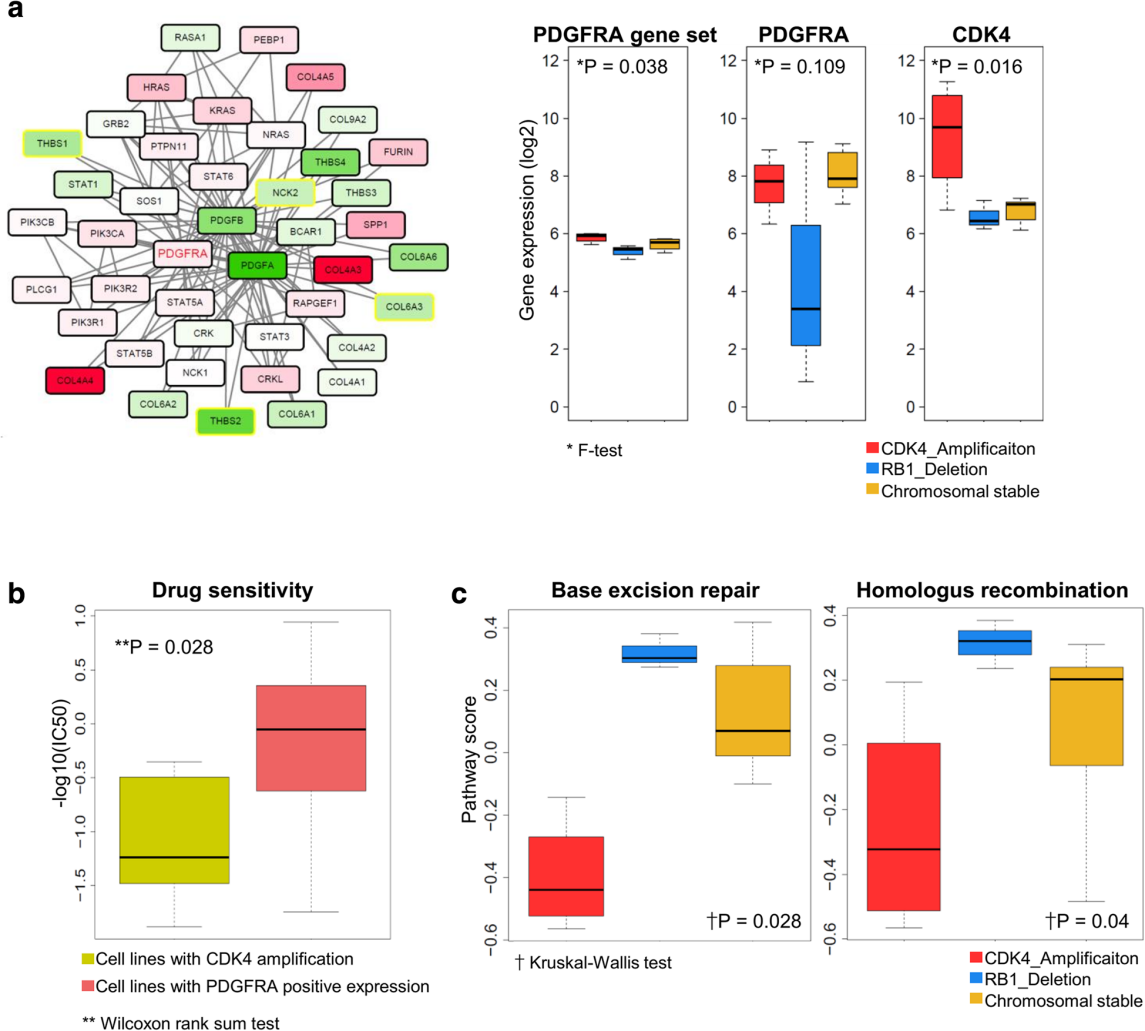
PDGFRA signaling pathway and drug sensitivity. **a** The sub-network that interacts with *PDGFRA* of the PDGF signaling pathway from REACTOME. Red or green nodes indicate up- or down-regulated genes in the *PDGFRA*-positive group compared with the *CDK4* amplification group. Yellow border colors of nodes indicate significant differential gene expression between the *PDGFRA*-positive group and the *CDK4* amplification group (*P* < 0.05; *t*-test). Edges between the nodes indicate the signaling interactions. Box plot of the average gene expression values (y-axis; *PDGFRA* sub-network, *PDGFRA* and *CDK4*, respectively) across samples within the sub-group (x-axis). P-values were calculated using the F-test. **b** Box plot of drug sensitivity (y-axis; -logIC50) in seven cell lines with *CDK4* amplification (pink) and four cell lines with *CDK4* normal copy number (green) across PDGFRA target drugs. P-values were calculated by the Wilcoxon rank-sum test. **c** Box plot of scores (y-axis) of pathways that enriched RB1 deletion across samples by sub-group (x-axis). P-values were calculated by the Kruskal-Wallis test

To investigate the differences between *CDK4* amp and CS groups, we identified the *PDGFRA* target drug responses in nine sarcoma cell lines. We assumed that the cells, which have both *CDK4* amplification and *PDGFRA* expression, would nb1ot show a good performance for *PDGFRA* target drugs, as *CDK4* has a role downstream of the *PDGFRA* signaling pathway. Six drugs showed IC_50_ sensitivity using the Cancer Therapeutics Response Portal v2 [20]. Four cell lines with no *CDK4* amplification and positive expression of *PDGFRA* had lower IC_50_ values than seven cell lines that had *CDK4* amplification and positive expression of *PDGFRA* (*P* = 0.028, Wilcoxon rank-sum test; Fig. 4b). We found that the *PDGFRA* signaling pathway in sarcoma cells, which have *CDK4* amplification and *PDGFRA* expression, was disarranged by *CDK4* activation, with decreased effects of drugs targeting *PDGFRA*. Finally, the molecular characteristics of the CS group were defined as a *PDGFRA* putative target group.

## Discussion

Given the wide genetic heterogeneity of STS types, defining the specific subtype is crucial for improving outcomes. According to a study that analyzed TCGA sarcoma data involving 206 sarcomas of six types, the type-specific copy-number alterations and histologic nuclear pleomorphisms correlate with aneuploidy estimates. In this study, we identified therapeutic groups across three CKS types and investigated the cancer drivers targeted by recently proposed drugs. A network analysis of the CKS abnormalities revealed *PDGFRA* as a putative target group and suggested that multiple-drug therapy in CKS could function via *CDK4* inhibition.

Molecular subtyping of the CKS samples showed the clusters of *CDK4* amp, *RB1* del, and *PDGFRA* putative target groups. Although our sample size was small (*n* = 14), the proportion of *CDK4* amp and *RB1* del showed no significant differences between the CKS and TCGA sarcoma populations. *CDK4* was amplified in 21.4% of CKS patients and in 18.7% of TCGA sarcoma patients (*P* = 0.83, *t*-test; Fig. 3b). The *RB1* deletion occurred in 21.4% of CKS patients and 9.7% of TCGA sarcoma patients (*P* = 0.76, *t*-test; Fig. 3b). Furthermore, the mutation landscape, mutation frequency, and MSI-h phenotype observations were analogous between the CKS and TCGA sarcoma populations. These results suggest that the molecular characterization of CKS in our small patient population is informative regarding the specific genomic landscape of CKS.

We found that the well-established somatic drivers of gene mutation – *FRG1B, CDC27, TP53, ATRX,* and *PTEN –* were altered in 57, 43, 21, 21, and 21% of CKSs. *FRG1B* and *CDC27* were highly mutated in CKS samples, but in only 3 and 0.4%, respectively, of TCGA sarcoma samples. *FRG1B* is in the FSHD region gene family, which was reported as a fusion gene in acute lymphoblastic leukemia [31]. *CDC27* is a tumor suppressor and a core component of cell cycle progression and degradation of G1/mitotic checkpoint regulators [32]. In the TCGA study, *TP53* and *ATRX* were high-recurrence mutations [6]. *TP53* is a tumor suppressor that regulates cell division by keeping cells from growing and proliferating. Missense mutations in *TP53* may influence cancer phenotype and survival [33]. *ATRX* plays an essential role in normal development, helping to regulate the expression of other genes through a process known as chromatin remodeling [34]. Additionally, long telomeres are associated with *ATRX* deletion or mutations in UPS and MFS [6, 35]. Somatic mutations in *PTEN* are known to cause tumorigenesis in human. A previous study suggested that the *PTEN* mutation might be a useful biomarker of cell proliferation in sarcomas [36].

The role of MSI-h in patients with CKS was suggested using mutation signature and expression values of MSI signature genes; MSI-h is found in 0–14% sarcoma patients [37]. Two studies suggested that patients with MSI-h sarcoma have inferior clinical outcomes [38, 39], but one study found no clinical correlation [40]. We identified two patients with MSI-h that had chromosomal stability, *PTEN* mutation, and low *PDGFRA* expression; one of these patients was classified as US. These results suggest that MSI-h is not a major phenotype in CKS, but could respond to immunotherapy. A phase II study for ipilimumab has been evaluated in sarcoma [41]. The FDA approved pembrolizumab (Keytruda) treatment for adult and pediatric patients with MSI-h, and the phase 2 trial showed encouraging activity in patients with UPS or dedifferentiated LPS [42]. Although the MSI-h phenotype in CKS remains controversial, the MSI-h state in CKS could be a therapeutic target for patient therapy.

To identify the molecular characteristics, a co-expression network was constructed using the relationship between gene copy number and gene expression profiles. Thirteen sub-network modules were identified, and the RB tumor suppressor-related pathway and PDGF receptor signaling network pathways were enriched (Additional file 1: Figure S5 c,d). The CS group was functionally classified into the *PDGFRA* putative target group. Recently, LARTRUVO (olaratumab), a *PDGFRA-*blocking antibody for anti-tumor activity against the PDGFRA signaling pathway, received FDA approval for STS therapy [43]. The median overall survival (OS) was improved by 11.8 months in patients who received LARTRUVO-doxorubicin compared to those who received doxorubicin alone. *CDK4* was significantly amplified in the CKSs and TCGA sarcoma patients. *CDK4*, a key molecule in the cell cycle, is associated with favorable prognosis in sarcoma [44]. Two LMS samples (LT01 and LT03) and two US samples (UT02 and UT03) were clustered in the *CDK4* amplification group, characterized by high expression of *PDGFRA, CDK4,* and *RB1*. The *CDK4* amp and CS groups showed high *PDGFRA* expression. Of note, *CDK4* amplification in sarcoma cells was not susceptible to the PDGFRA target drug. Although PDGFRA inhibition can occur in CKS, tumorigenesis is enhanced by *CDK4* amplification, which is a role downstream of the PDGFRA signaling pathway. High-level *CDK4* amplification is associated with poor recurrence-free survival compared to low-level *CDK4* amplification [45]. Palbociclib treatment was associated with a good progression-free rate in patients with *CDK4*-amplification and *RB* expression [46]. Furthermore, the *CDK4* amp group was enriched in both the CKSs and TCGA sarcomas in patients with recurrence. These indications show that *CDK4*-targeted therapies in sarcomas can be used across CKS types; continuous monitoring for disease recurrence is required. The *RB1* del group was identified in three CKS samples (LT02, LT04, and LT06). In the network module and gene set enrichment analysis, the RB tumor suppressor-related pathway and DNA damage repair system related pathways such as DNA replication and homologous recombination was enriched in the RB1 del group (Additional file 1: Figure S4). RB1 loss leads to tumorigenesis, with regulation of the DNA damage repair system. In particular, the loss of RB1 occurs in angiosarcomas and osteosarcomas, supporting a role for tumor suppressor in pathogenesis [47]. Recently, taxane chemotherapy (cabazitaxel) has been suggested in *RB1*-depleted tumor therapy [48], and used as an FDA-approved prostate cancer treatment. Phase II trial for cabazitaxel antitumor activity in LPS is in progress (NCT01913652). Our findings suggest that CKSs can be classified into three therapeutic subgroups; cancer drivers for each group should be targeted in CKS therapies.

We investigated 74 drug responses in nine sarcoma cell lines with available data to verify the applicability of cancer therapies with subgroups. We identified the responses of three drugs (sunitinib, JQ1, and UNC0638) in five *RB1* del type cell lines (Additional file 1: Figure S6). Sunitinib is a multi-targeted receptor tyrosine kinase (e.g. PDGFR) inhibitor that was approved by the FDA for the treatment of renal cell carcinoma and gastrointestinal stroma tumors. JQ1 is a small molecule that inhibits the BET family [49]. Although this molecule awaits approval for marketing, it may be considered as a potential drug for patients with RB1 deletion. UNC0638, a type of small molecule that can regulate the activity of histone methyltransferase [50], has been reported as a promising drug for cancer therapy [51].

## Conclusions

This study had several limitations, such as the small number of patients with CKS. Additionally, this retrospective study included patients having one of three types of CKS, so the results are not generalizable to a broader population of patients with CKS. However, the frequency of actionable mutations and SCNVs did not differ from the TCGA reports in large CKS population studies, which proposed three therapeutic subgroups. The patterns of molecular characterization revealed in our study may provide clues to the target medications in patients with CKS. Further subgroup classification protocols and targeted therapy in CKS will be required to analyze long-term clinical trials that may translate into therapeutic benefits for CKS patients with poor prognosis.

## Additional file

Additional file 1: Integrated molecular characterization of adult soft tissue sarcoma for therapeutic targets. **Figure S1.** Expression with sub-clusters of MSI status**.** Box plots for the average expression values with sub-clusters by NMF clustering. *P*-values were calculated using the *t*-test. **Figure S2.** SCNAs**.** Recurrent focal copy number alterations in 14 CKS samples by GISTIC2. Red and blue lines indicate the significant amplified and deleted regions, respectively. **Figure S3.** Somatic copy number alteration (SCNA) clustering in 206 TCGA sarcoma data. Hierarchical clustering using the copy number profiles of focal regions (7p22.3; CDK4, 1q21.2, 13q14., and 12q14.1; RB1). **Figure S4.** Pathway analysis using gene expression**.** A heat map indicates the scores from the GSEA analysis. Euclidean clustering was performed on the KEGG pathway. FT, Myxofibrosarcoma; LT, Leiomyosarcoma; UT, Undifferentiated sarcoma. **Figure S5.** Network analysis of significant genes correlated with expression and copy number. ***(a)*** Heat map of the 26 genes showing a significant relationship between copy number and gene expression profiles. ***(b)*** Whole network of 556 genes and 11,643 interactions, including the 26 genes and their first neighbors. Red or blue nodes indicate amplified or deleted, respectively. Border colors of nodes indicate expression values. ***(c,d)*** Sub-modules of RB tumor suppressor-related and PDGFRA receptor signaling pathway, respectively. **Figure S6.** Drug responses in cell lines with RB1 del type. Box plot of drug sensitivity (y-axis; area under the dose-response curve) in five cell lines with RB1 del (dark blue) and 11 other sarcoma cell lines. P-values were calculated by *t*-test. (DOCX 923 kb)

## Abbreviations

CDK4: Cyclin-dependent kinase 4
CKS: Complex karyotype sarcoma
LMS: Leiomyosarcoma
LPS: Liposarcoma
MFS: Myxofibrosarcoma
MSI: Microsatellite instability
NMF: Non-negative matrix factorization
PDGFRA: Platelet-derived growth factor receptor A
RB1: Retinoblastoma 1
SCNA: Somatic copy number alteration
US: Undifferentiated sarcoma
