# Supplementary material for: Integrated molecular characterization of adult soft tissue sarcoma for therapeutic targets
Source: BMC Med Genet. 2018 Dec 31;19(Suppl 1):216. doi: 10.1186/s12881-018-0722-6 (PMC6311917; doi:10.1186/s12881-018-0722-6)

**Integrated molecular characterization of adult soft tissue sarcoma for therapeutic targets**

**Jihyun Kim^1*^, June Hyuk Kim^2*^, Hyun Guy Kang^2, 3^, Seog Yun Park^4^, Jung Yeon Yu^5^, Eun Young Lee^5^, Sung Eun Oh^2^, Young Ho Kim^6^, Tak Yun^6^, Charny Park^1^, Soo Young Cho^1†^ and Hye Jin You^3,5†^**

^1^Clinical Genomic Analysis Branch, Research Institute, National Cancer Center; ^2^Orthopaedic Oncology Clinic, Hospital, National Cancer Center; ^3^Department of Cancer Biomedical Science, NCC-GCSP, National Cancer Center; ^4^Division of Pathology, Hospital, National Cancer Center; ^5^Translational Research Branch, Research Institute, National Cancer Center; ^6^Rare Cancer Branch, Research Institute, National Cancer Center

**^†^**Corresponding to: Hye Jin You & Soo Young Cho

Hye Jin You

Translational Research Branch, Research Institute; Department of Cancer Biomedical Science, National Cancer Center-Graduate School of Cancer Science and Policy

323 Ilsan-ro, Ilsandong-gu, Goyang, Gyeonggi, 10408 South Korea.

Email:hjyou@ncc.re.kr

Soo Young Cho

Clinical Genomic Analysis Branch, Research Institute, National Cancer Center

323 Ilsan-ro, Ilsandong-gu, Goyang, Gyeonggi, 10408 South Korea.

Email:sooycho@ncc.re.kr

*authors contribute equally

**Additional File Legend**

**Fig. S1.** Expression with sub-clusters of MSI status**.** Box plots for the average expression values with sub-clusters by NMF clustering. P-values were calculated using the *t*-test.

**Fig. S2.** SCNAs**.** Recurrent focal copy number alterations in 14 CKS samples by GISTIC2. Red and blue lines indicate the significant amplified and deleted regions, respectively.

**Fig. S3.** Somatic copy number alteration (SCNA) clustering in 206 TCGA sarcoma data. Hierarchical clustering using the copy number profiles of focal regions (7p22.3; CDK4, 1q21.2, 13q14., and 12q14.1; RB1).

**Fig. S4.** Pathway analysis using gene expression**.** A heat map indicates the scores from the GSEA analysis. Euclidean clustering was performed on the KEGG pathway. FT, Myxofibrosarcoma; LT, Leiomyosarcoma; UT, Undifferentiated sarcoma.

**Fig. S5.** Network analysis of significant genes correlated with expression and copy number.  ***(a)*** Heat map of the 26 genes showing a significant relationship between copy number and gene expression profiles. ***(b)*** Whole network of 556 genes and 11,643 interactions, including the 26 genes and their first neighbors. Red or blue nodes indicate amplified or deleted, respectively. Border colors of nodes indicate expression values. ***(c,d)*** Sub-modules of RB tumor suppressor-related and PDGFRA receptor signaling pathway, respectively.

**Fig. S6.** Drug responses in cell lines with RB1 del type. Box plot of drug sensitivity (y-axis; area under the dose-response curve) in five cell lines with RB1 del (dark blue) and 11 other sarcoma cell lines. P-values were calculated by *t*-test.


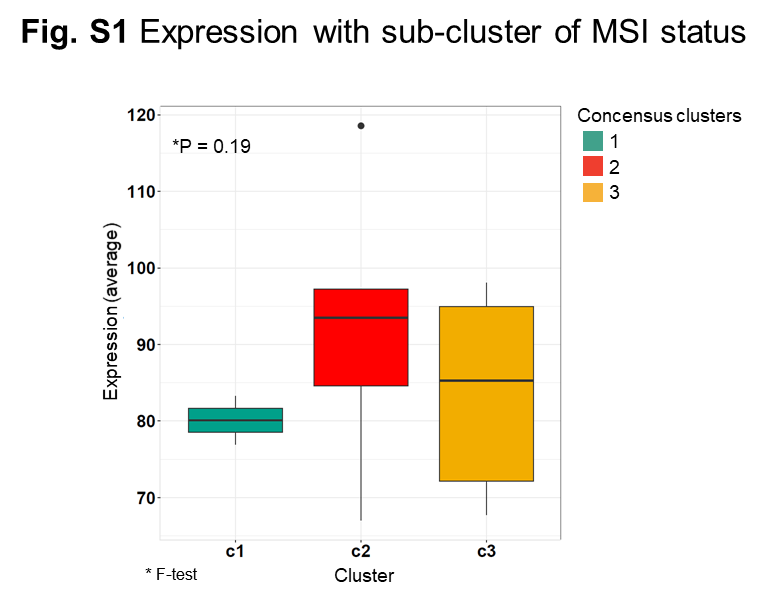


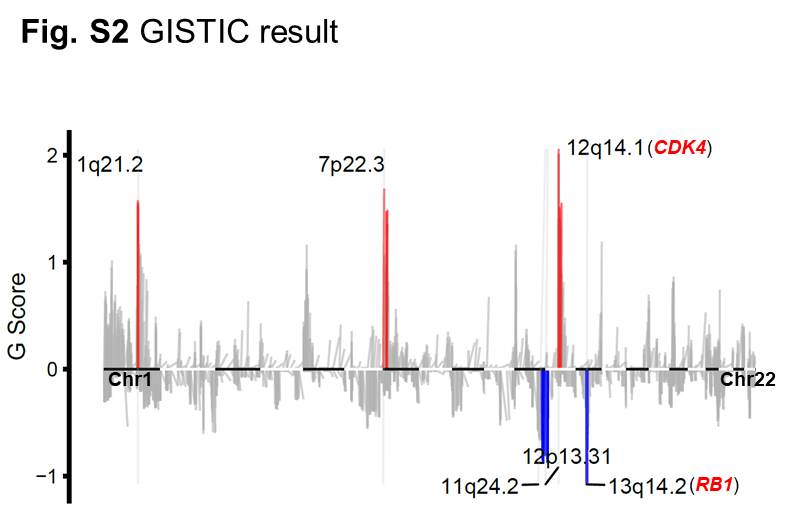


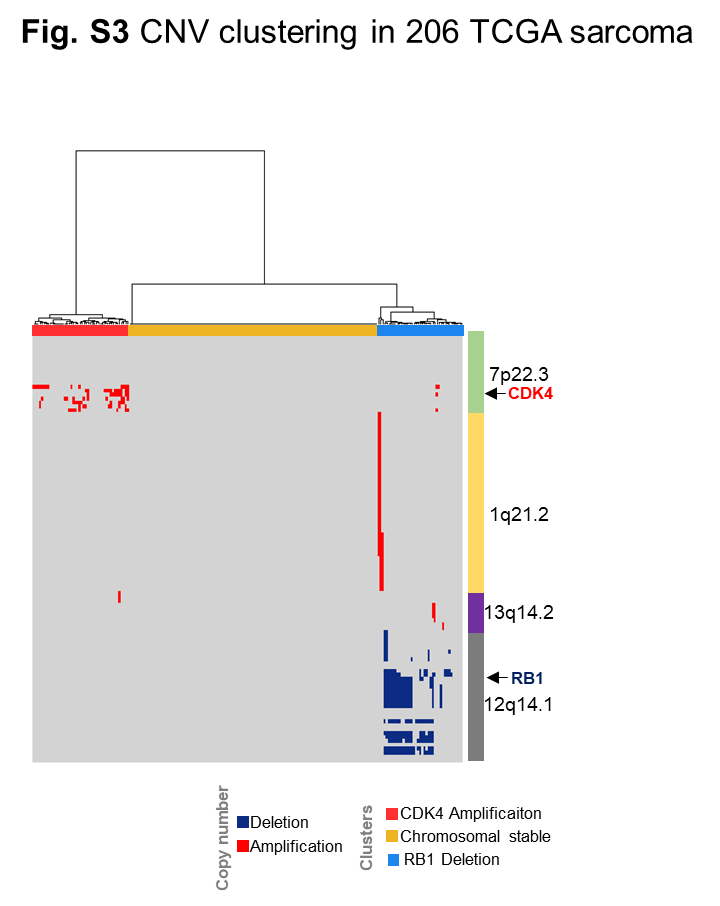


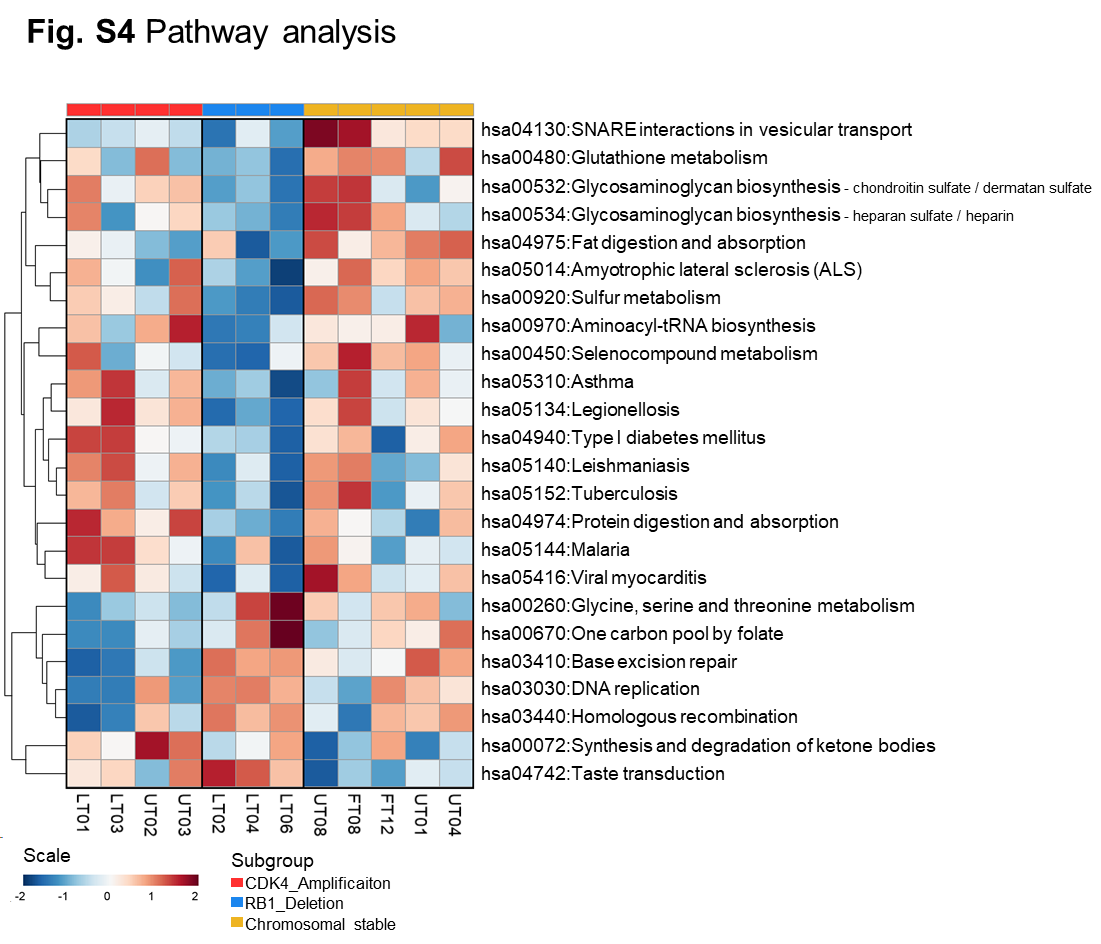


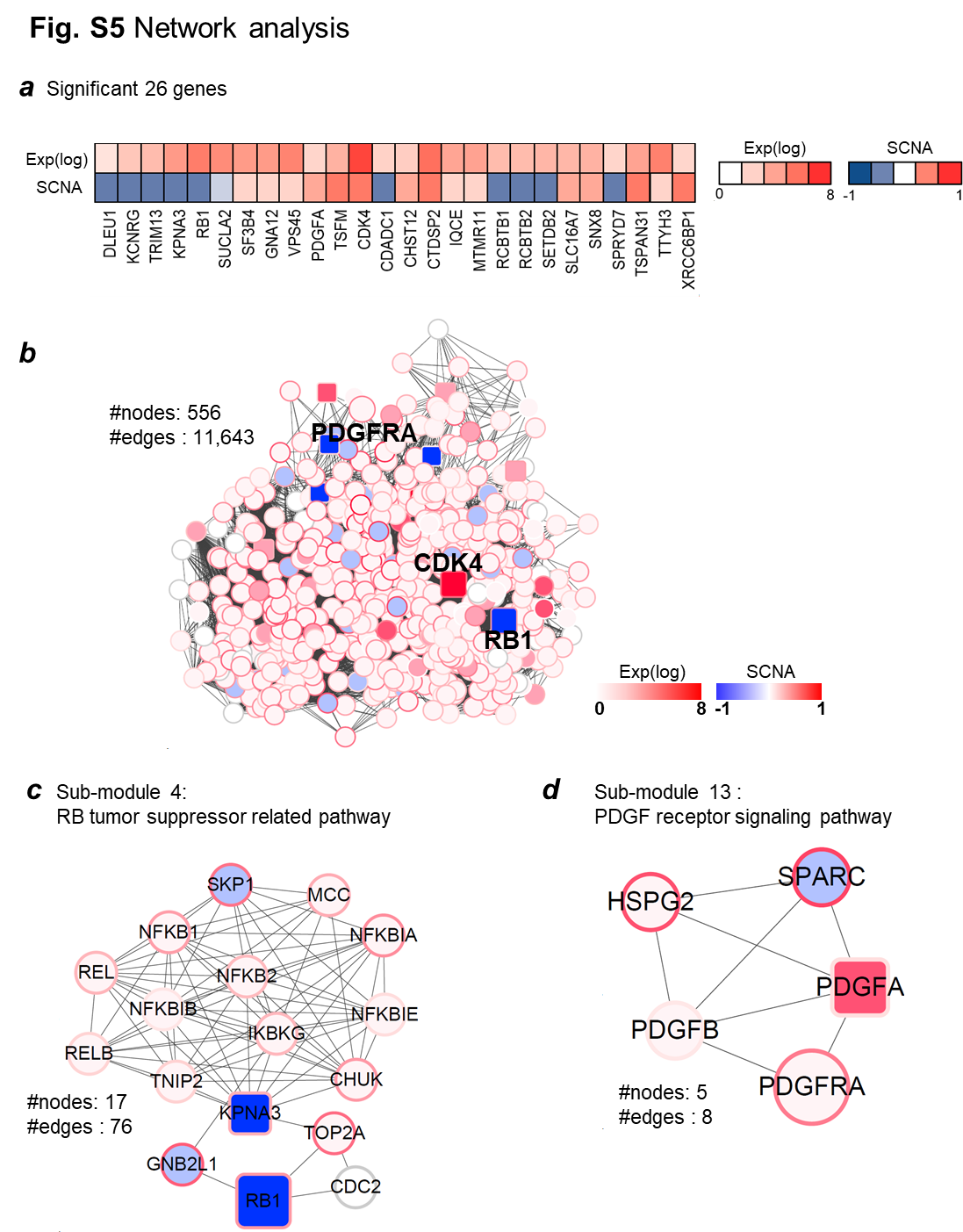


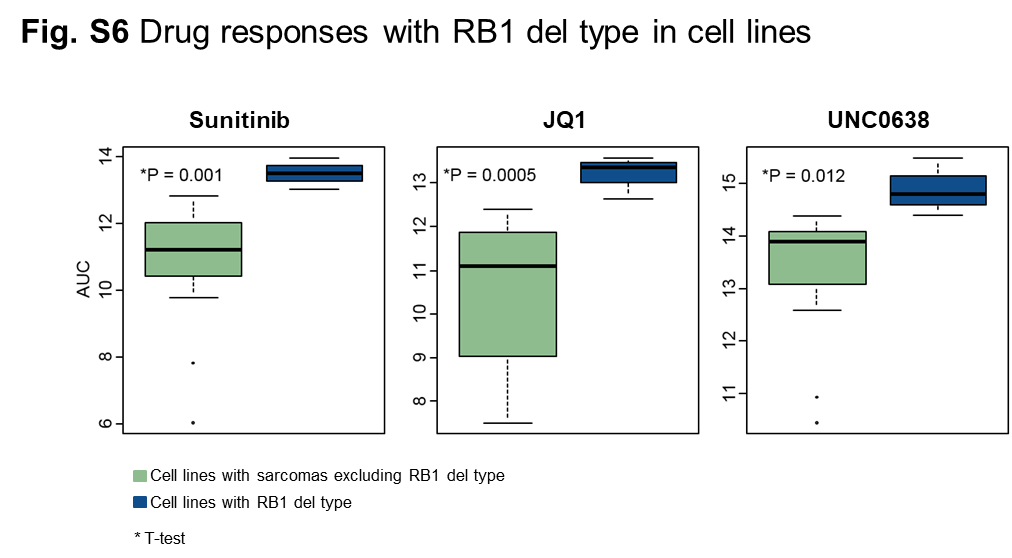

Supplement: Supplementary file 1 — Integrated molecular characterization of adult soft tissue sarcoma for therapeutic targets. Figure S1. Expression with sub-clusters of MSI status. Box plots for the average expression values with sub-clusters by NMF clustering. P-values were calculated using the t-test. Figure S2. SCNAs. Recurrent focal copy number alterations in 14 CKS samples by GISTIC2. Red and blue lines indicate the significant amplified and deleted regions, respectively. Figure S3. Somatic copy number alteration (SCNA) clustering in 206 TCGA sarcoma data. Hierarchical clustering using the copy number profiles of focal regions (7p22.3; CDK4, 1q21.2, 13q14., and 12q14.1; RB1). Figure S4. Pathway analysis using gene expression. A heat map indicates the scores from the GSEA analysis. Euclidean clustering was performed on the KEGG pathway. FT, Myxofibrosarcoma; LT, Leiomyosarcoma; UT, Undifferentiated sarcoma. Figure S5. Network analysis of significant genes correlated with expression and copy number. (a) Heat map of the 26 genes showing a significant relationship between copy number and gene expression profiles. (b) Whole network of 556 genes and 11,643 interactions, including the 26 genes and their first neighbors. Red or blue nodes indicate amplified or deleted, respectively. Border colors of nodes indicate expression values. (c,d) Sub-modules of RB tumor suppressor-related and PDGFRA receptor signaling pathway, respectively. Figure S6. Drug responses in cell lines with RB1 del type. Box plot of drug sensitivity (y-axis; area under the dose-response curve) in five cell lines with RB1 del (dark blue) and 11 other sarcoma cell lines. P-values were calculated by t-test. (DOCX 923 kb) [file 12881_2018_722_MOESM1_ESM.docx]
